# Detailed Assessment of Acrel Ganglion Through Cadaveric Dissection, Histology, and Ultrasonography

**DOI:** 10.7759/cureus.94388

**Published:** 2025-10-12

**Authors:** Saima Khan, Attka Maryam, Qurrat ul ain Aamir, Shakeela Nazir, Chinedu J Enwerem, Haider Hilal

**Affiliations:** 1 School of Medicine, St. George's University School Of Medicine, Newcastle Upon Tyne, GBR; 2 Radiology, Rashid Latif Medical College, Lahore, PAK; 3 Radiology, Shaukat Khanum Memorial Cancer Hospital and Research Centre, Lahore, PAK; 4 Department of Anatomy, Rahbar Medical and Dental College, Lahore, PAK; 5 Department of Anatomy, Rashid Latif Khan University Medical College, Lahore, PAK; 6 Anatomical Sciences, St. George's University School of Medicine, Newcastle Upon Tyne, GRD; 7 School of Medicine, St. George's University School of Medicine, Newcastle Upon Tyne, GBR

**Keywords:** acrel’s pseudo-ganglion, ganglion, posterior interosseus, radial nerve entrapments, ultrasound

## Abstract

Background

Acrel’s “pseudo-ganglion” is a ganglioform structure found on the floor of the fourth dorsal extensor compartment. The function of Acrel’s “pseudo-ganglion” is undetermined within published literature; however, recent histological studies suggest it to be devoid of nerve cell bodies and is, therefore, a “pseudo-ganglion." Advances in ultrasound technology have made it possible to better visualize nerves in vivo. This study aims to investigate any ganglioform structure at the termination of the posterior interosseous nerve, using ultrasound, cadaveric dissection, and histological studies.

Materials and methods

Twenty wrists from 10 formalin-fixed cadavers were dissected and examined. Standard haematoxylin and eosin staining of cadaveric samples was performed to check for the presence of any ganglionic cells and other cellular organizations. Ultrasound examination of the wrists of 10 live humans, using a GE LOGIQ e ultrasound system manufactured by GE Healthcare at Chicago, USA, with a 12L-RS transducer, was performed, looking for a non-compressible hypoechoic ovoid structure on the lateral side of the fourth extensor compartment, medial to Lister’s tubercle in accordance with the described location of Acrel’s “pseudo-ganglion."

Results

A ganglioform swelling was found in all cadavers, which corresponded to a non-compressible hypoechoic ovoid structure under ultrasonography, on the radial/lateral side of the fourth extensor compartment, and ulnar/medial to the dorsal/Lister’s tubercle. Standard haematoxylin and eosin staining from cadaveric samples showed the absence of any neuronal cells and inflammatory cells.

Conclusion

Understanding the termination of the posterior interosseous nerve under different modalities enabled us to gain knowledge about Acrel’s “pseudo-ganglion,” and this can aid in the assessment of various wrist pathologies linked to tendons around the wrist and their relation to this ganglioform swelling, named Acrel anglion. It may also help in refining clinical applications, being minimally invasive through the use of ultrasound and targeting that precise location.

## Introduction

The posterior interosseous nerve is the principal nerve of the posterior compartment of the forearm, supplying the majority of the extensor muscles and those of the deep layer [1,2]. Distally, it continues toward the wrist joint, where it may demonstrate a localized fusiform enlargement. This structure has historically been described as Acrel’s pseudo-ganglion, named after the 18th-century Swedish surgeon Olof af Acrel (1717-1806). Although recognized for centuries, the functional significance of this swelling remains uncertain. It has been proposed to represent a distal expansion from which articular branches arise, with a potential proprioceptive role; however, histological studies have not confirmed the presence of neural cell bodies within the enlargement, supporting its designation as a “pseudo-ganglion” [1].

Ultrasound is a non-invasive, widely available imaging modality capable of demonstrating real-time static and dynamic features of peripheral nerves and their relationships to surrounding anatomical structures. Advances in high-resolution ultrasound technology have led to increased applications in the evaluation of soft-tissue lesions and nerve pathologies in the wrist and hand [3,4].

Accurate identification of the posterior interosseous nerve in the distal forearm and an improved understanding of its sonographic features are valuable for evaluating potential nerve involvement in wrist disorders [4,5]. Such knowledge may also aid in the development of diagnostic and therapeutic ultrasound-guided interventions targeting the nerve and its terminal branches. The present study aims to assess the feasibility of identifying Acrel’s pseudo-ganglion in vivo using ultrasound and to characterize its anatomical relationship to the posterior interosseous nerve.

## Materials and methods

Ethical approval and study design

This cadaveric and clinical anatomical study was conducted over a two-month period following approval from the Ethical Review Committee at Rashid Latif Khan University Medical College (IRB reference number: IRB/2018/005). Both cadaveric dissections and ultrasonographic evaluations were performed to investigate the posterior interosseous nerve (PIN) in relation to Acrel’s pseudo-ganglion.

Cadaveric dissections

**Figure 1:**
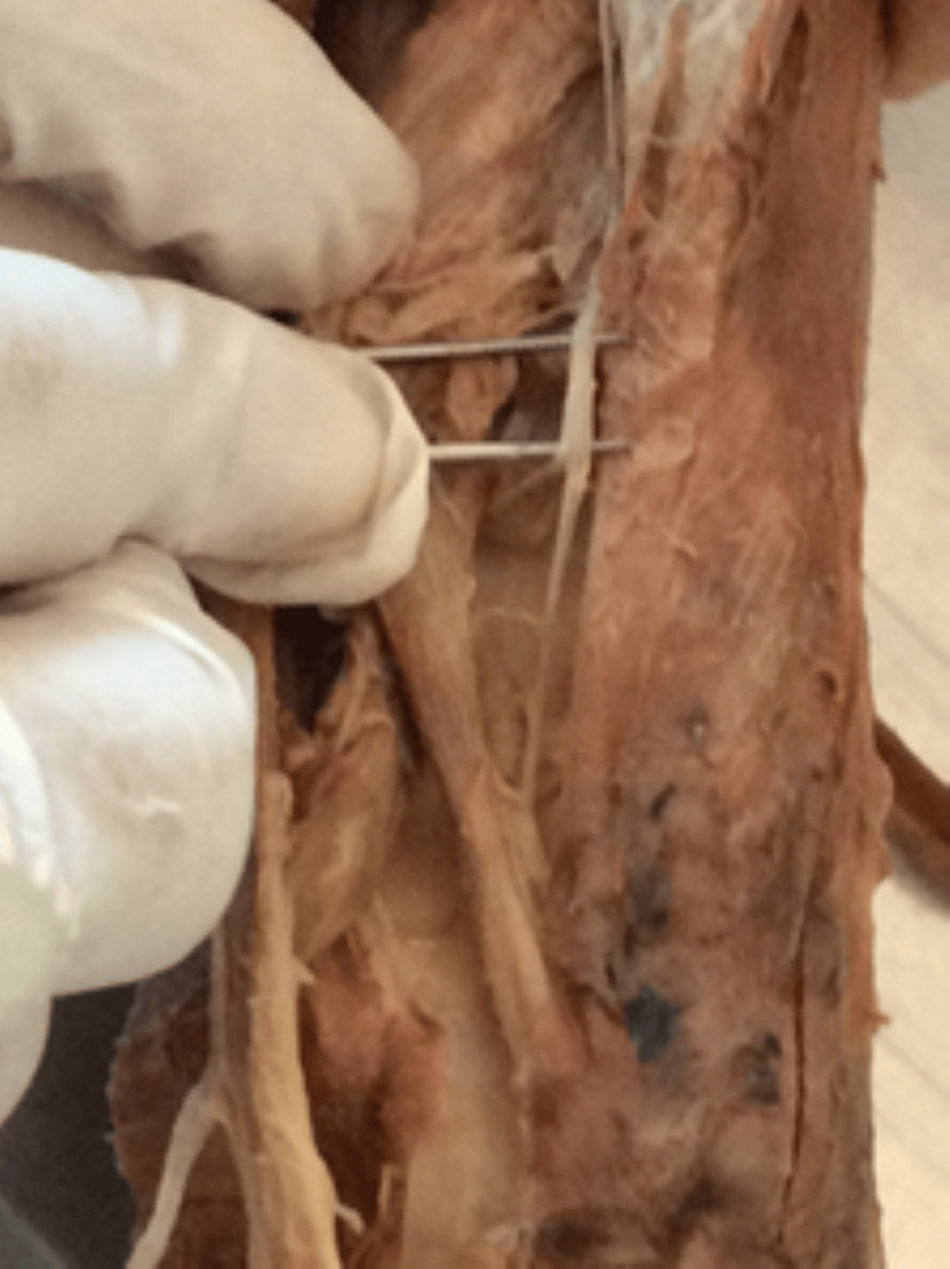
Cadaveric dissection of posterior interosseous nerve, showing a thickened band of nerve (above the forceps) near to the termination.

Twenty wrists from 10 formalin-fixed cadavers were included. Careful dissection of the dorsal forearm and wrist was undertaken to expose and trace the PIN distally towards its termination (Figure 1). Tissue samples were obtained from the region of interest for histological examination. Standard haematoxylin and eosin (H&E) staining was carried out to assess the presence of ganglionic cells and evaluate overall tissue organization.

Ultrasonographic examination

The in vivo component comprised 10 wrists from five healthy adult participants. Inclusion criteria required participants to have no prior history of wrist trauma, surgery, or deformity of the distal forearm and wrist.

**Figure 2:**
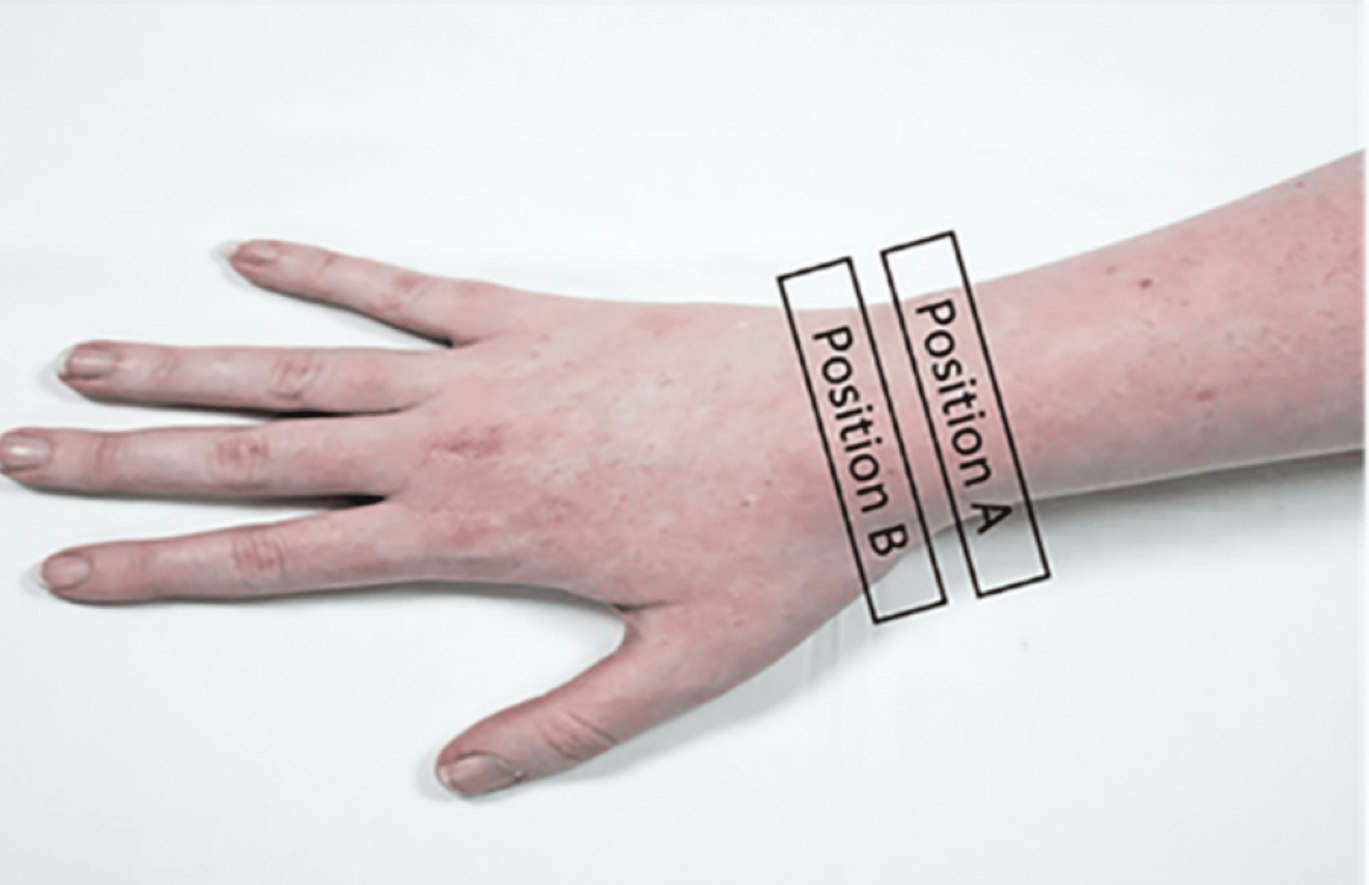
Position A and Position B used as a landmark to visualise termination of the posterior interosseous nerve.

Ultrasonographic evaluation was performed using a GE LOGIQ e ultrasound system (GE Healthcare, Chicago, USA) with a 12L-RS linear transducer. Participants were seated with their forearms resting on a flat surface. Transverse, short-axis images were obtained at the dorsal wrist, with attention to the fourth dorsal extensor compartment and the dorsal/Lister’s tubercle (Figure 2). Upon identifying the presumed Acrel’s pseudo-ganglion, the transducer was moved proximally to trace the PIN and examine its continuity or connection with the pseudo-ganglion.

Data collection and analysis

Data were collected from two sources: ultrasonographic images of living participants and histological specimens from cadaveric dissections. Ultrasonographic images were analyzed to define the anatomical course of the PIN and its relationship to the pseudo-ganglion. Histological slides were reviewed to detect ganglionic cells and describe structural features. The findings from cadaveric and ultrasonographic components were compared to establish concordance between anatomical, imaging, and histological observations.

## Results

**Figure 3:**
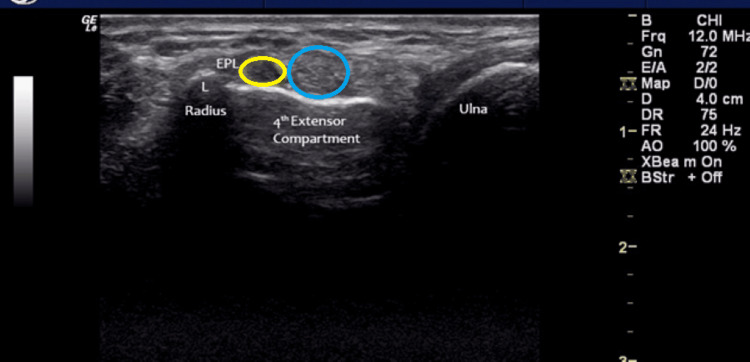
Extensor pollicis longus tendon (yellow circle) and termination of the posterior interosseous nerve (blue circle).

During ultrasonographic assessment, a distinct, non-compressible, hypoechoic ovoid structure was consistently visualized on the radial (lateral) aspect of the fourth extensor compartment, positioned ulnar (medial) to the dorsal bony prominence known as Lister’s tubercle, in all wrists examined (Figure 3). The structure was readily identifiable in both longitudinal and transverse imaging planes, permitting accurate delineation of its morphology and spatial relationship to adjacent anatomical structures, including the extensor tendons and the underlying carpal cortex. Its superficial location relative to the dorsal carpal surface facilitated consistent visualization across examinations, thereby confirming its reproducible anatomical position.

The anatomical localization of this hypoechoic ovoid structure corresponds closely with the site historically described as Acrel’s pseudo-ganglion. Sonographically, the structure exhibited uniform hypoechogenicity and a smooth, well-defined contour, distinct from the typical sonographic characteristics of peripheral nerves. Whereas peripheral nerves generally display a hyper-echoic perineurial boundary and a honeycomb internal architecture, attributable to alternating fascicular and interfascicular connective tissue patterns, the observed structure lacked this fascicular organization. The absence of a honeycomb pattern and its homogeneous echotexture suggest a non-fascicular composition, supporting the hypothesis that Acrel’s pseudo-ganglion may represent a localized fibrous or connective tissue modification rather than a true ganglionic or cystic entity.

The posterior interosseous nerve was identified proximal to the dorsal tubercle/Lister’s tubercle, demonstrating the typical honeycomb/hyper-echoic characteristic of a nerve on ultrasound scans (Figure 3). It was possible to determine a connection between Acrel’s pseudo-ganglion and the posterior interosseous nerve by tracing the nerve proximally in the forearm.

**Figure 4:**
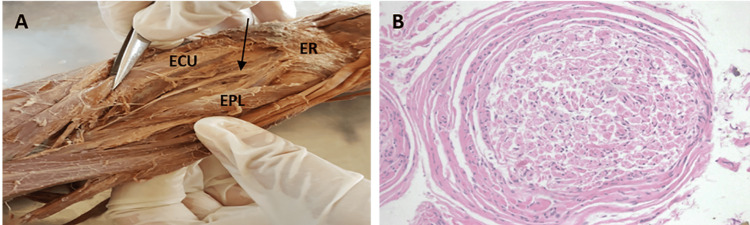
A: Cadaveric image of the PIN. B: Histology of the termination of PIN. PIN: posterior interosseous nerve, ECU: extensor carpi ulnaris, ER: extensor retinaculum, EPL: extensor pollicis longus

After detailed dissection of the posterior interosseous nerve, a histological examination of the structure, shown in Figure 4A (arrow), was performed and revealed no inflammatory response in these histologically normal peripheral nerve structures. No neuronal cell bodies were identified (Figure 4B). 

## Discussion

Pseudo-ganglion or gangliform enlargements have been described in association with several nerves of the upper and lower limbs [6,7,8], including the lateral femoral cutaneous, plantar connecting branch, nerve to teres minor, posterior interosseous, and accessory obturator nerves [9-13]. The etiology and function of these enlargements remain largely undetermined; however, anatomical studies have suggested that mechanical friction or pressure could contribute to increased thickening of the connective tissue encasement surrounding these nerves [13].

In their study, Edelson and Nathan reported finding an enlargement in the lateral femoral cutaneous nerve as it passes under the inguinal ligament in 51% of the cases investigated [10]. No pseudoganglia were found in fetal specimens, suggesting that a combination of erect posture and the course of the nerve underneath the inguinal ligament may cause mechanical friction and irritation. The authors also proposed that the pseudo-ganglion of the lateral femoral cutaneous nerve could play a role in idiopathic meralgia paresthetica.

Arakawa et al. reported evidence of thickened connective tissue encasement surrounding the connecting branch between the deep branch of the lateral plantar nerve and the medial plantar nerve in all 19 cases where this connecting branch was present [11]. They also suggested that the location of this branch between the tendons of flexor hallucis longus and flexor hallucis brevis makes it vulnerable to pressure or friction during gait [11]. The authors highlighted a potential relationship between the presence of a pseudo-ganglion in this connecting branch and pressure neuropathies of the foot.

Other anatomical studies have also reported pseudoganglia associated with the nerve to teres minor [12], the terminal branch of the deep fibular nerve and the accessory obturator nerve [13], attributing their formation to mechanical friction and irritation by the tendon of triceps brachii, the extensor digitorum brevis muscle, and the pelvic brim, respectively.

Tubbs et al. found no histological signs of chronic irritation or thickening of the epineurium surrounding Acrel’s pseudo-ganglion of the posterior interosseous nerve [13]. Similarly, all wrists investigated in their study were from asymptomatic participants without deformities in the wrist area. Based on these findings and other reports in the literature [8], the etiology of Acrel’s pseudo-ganglion remains unclear, and the mechanical friction hypothesis cannot be supported.

In an ultrasound study of the posterior interosseous nerve, Acrel’s pseudo-ganglion was not visualized by ultrasound or during subsequent dissections [14]. However, in this study, a non-compressible hypoechoic ovoid structure at the radial/lateral side of the fourth extensor compartment, ulnar/medial to the dorsal (Lister’s) tubercle, corresponding to the described location of Acrel’s pseudo-ganglion, was identified in all wrists. The posterior interosseous nerve was also visualized proximal to Lister’s tubercle, showing the typical honeycomb/hyperechoic appearance on ultrasound scans. Histological study confirmed no ganglion cells within the swelling, supporting its classification as a pseudo-ganglion. These findings suggest that Acrel’s pseudo-ganglion can be visualized by ultrasound and differentiated from the posterior interosseous nerve. Further studies may help establish protocols for optimal visualization of the posterior interosseous nerve and Acrel’s pseudo-ganglion, considering different transducers, ultrasound systems, and inter-observer variation [14,15].

Several limitations should be considered when interpreting the findings from the available literature on pseudo-ganglia. First, many of the anatomical studies are based on small sample sizes, often limited to cadaveric dissections, which restricts the generalizability of their observations. Second, most reports are descriptive in nature, and few provide quantitative analyses of the prevalence, size, or histological features of pseudo-ganglia. Third, there is a lack of longitudinal or clinical correlation data, making it difficult to establish a causal relationship between pseudo-ganglion formation and symptomatic neuropathies such as meralgia paresthetica or plantar pressure neuropathies. Additionally, variability in dissection techniques, imaging modalities, and interpretation across studies may contribute to inconsistencies in reported findings. Finally, the absence of standardized diagnostic criteria for pseudo-ganglia complicates comparisons between studies and limits the development of a unified understanding of their etiology and clinical significance.

## Conclusions

Understanding the sonographic properties of the posterior interosseous nerve and Acrel’s pseudo-ganglion can aid in the proper assessment of nerve involvement in various wrist pathologies. It may also help in developing novel clinical applications that are minimally invasive through the use of ultrasound. Moreover, the ability to characterize these structures with high-resolution imaging can improve diagnostic accuracy, assist in differentiating them from other soft tissue lesions, and reduce the likelihood of unnecessary invasive procedures.

Given its accessibility and cost-effectiveness, ultrasound may serve not only as a first-line tool for evaluation but also as a valuable modality for guiding targeted interventions such as injections or minimally invasive decompressions. In addition, routine sonographic evaluation may facilitate early detection of subtle changes, enabling timely management and potentially better clinical outcomes.

Future research focusing on standardizing ultrasound protocols and validating findings with surgical or histopathological correlations will further strengthen its role in clinical practice. Ultimately, expanding the use of ultrasound in this context highlights its promise as both a diagnostic and therapeutic tool, bridging the gap between imaging and intervention in wrist pathologies.
